# Synbiotic containing extensively hydrolyzed formula improves gastrointestinal and atopic symptom severity, growth, caregiver quality of life, and hospital‐related healthcare use in infants with cow's milk allergy

**DOI:** 10.1002/iid3.636

**Published:** 2022-05-19

**Authors:** Gary P. Hubbard, Kiranjit Atwal, Lynne Graham, Sankara Narayanan, Lisa Cooke, Catherine Casewell, Sally‐Ann Denton, Joan Gavin, Robert M. Browne, Fiona J. Kinnear, Ailsa J. McHardy, Debbie Evans, Rachel Vallis, Devasmitha Venkataraman, Abbie L. Cawood, Sarah Donohoe, Victoria Steele, Sonia Armstrong, Rebecca J. Stratton

**Affiliations:** ^1^ Nutricia Ltd Trowbridge UK; ^2^ West Hertfordshire Hospitals NHS Trust Watford UK; ^3^ Bristol Royal Hospital for Children Bristol UK; ^4^ Ashford and St Peter's Hospital NHS Foundation Trust Chertsey UK; ^5^ University Hospitals Southampton NHS Foundation Trust Southampton UK; ^6^ Royal Surrey NHS Foundation Trust Guildford UK; ^7^ James Cook Hospital, South Tees Hospitals NHS Foundation Trust Middlesbrough UK; ^8^ Faculty of Medicine University of Southampton Southampton UK

**Keywords:** cow's milk allergy, dermatitis, prebiotics, probiotics, synbiotics

## Abstract

**Background:**

Healthy gut microbiota is important for prognosis in cow's milk allergy (CMA). The application of synbiotics (specific pre‐ and probiotics) in extensively hydrolyzed formulae (eHFs) is a relatively new concept.

**Aims:**

To evaluate a synbiotic‐containing, whey‐based eHF (SeHF) with galacto‐oligosaccharides, fructo‐oligosaccharides, and bifidobacterium breve M‐16V in infants with CMA.

**Materials and Methods:**

A 31‐day one‐arm pilot study in 29 infants with CMA (mean age 30.8 weeks [SD 11]) was undertaken, with outcomes including gastrointestinal tolerance, atopic dermatitis symptoms, dietary intake, growth, SeHF acceptability, caregiver quality of life, and hospital‐related healthcare use.

**Results:**

Significant improvements (*p* < .05) in the severity of abdominal pain (in 57%), burping (in 46%), flatulence (in 79%), constipation (in 14%), rhinitis (41%), and itchy eyes (73%), as well as atopic dermatitis in those with severe baseline symptoms (PO‐SCORAD^©^ reduction: 34.7–18.2 (*p* = .003), *n* = 6) were observed over time. Growth and caregiver quality of life scores significantly increased (+26.7%, *p* < .05) over time. Hospital visits and medications significantly reduced (−1.61 and −2.23, respectively, *p* < .005) in the 6 months after SeHF initiation.

**Discussion:**

In this small, single‐arm, pilot study, the use of SeHF enhanced the management of infants with non‐IgE mediated CMA who were already established on eHF. Conclusion: Whilst this study adds to the evidence base for the use of SeHF in CMA, further robust research to explore the longer‐term benefits of synbiotics, specifically the blend used in this study, for the clinical management of infants with CMA is warranted.

## Graphical abstract

The application of synbiotics (specific pre‐ and probiotics) in extensively hydrolyzed formula (SeHF) is a relatively new concept. This study investigated the use of SeHF in 29 infants with non‐IgE mediated Cow's Milk Allergy and showed improvements in gastrointestinal and atopic symptoms, caregiver quality of life, and reduced healthcare use. The use of SeHF enhanced the management of infants with non‐IgE mediated cow's milk allergy

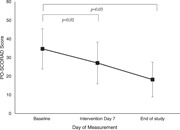

## 1 INTRODUCTION

Primordial development of the gut microbiome in infants represents a period of profound significance in the first few years of life, affecting immune function and inflammatory response.^1^, ^2^ Alterations in the gut microbiota during this stage may predispose to disease later in life. It is well established that allergic infants have a disordered microbiome (otherwise known as dysbiosis) with lower microbial diversity, increased presence of pathogenic gut microbiota species (*Clostridia* and *Coliform*), and reduced counts of beneficial gut microbiota species such as *Bifidobacterium* (specifically *Bifidobacterium breve* and *Bifidobacterium bifidum)* which precede the development of atopy.^3^, ^4^, ^5^, ^6^, ^7^ Development of healthy gut microbiota may assist in reducing atopic disease by improving gut barrier function, assisting gut‐associated lymphoid tissue, and promoting production of anti‐inflammatory and immunomodulatory substances.^3^, ^8^, ^9^ As such, there has been a great deal of interest in understanding the factors that modulate the gut microbiota of allergic infants. The impact of maternal microbiota, mode of infant delivery, exposure to antibiotics, and feeding practice (diet) in early life are some of the factors that have been identified.^10^

A strong body of evidence illustrates that dietary components such as pre‐ and probiotics modulate the gut microbiota. Prebiotics are defined as a nondigestible food ingredient such as galacto‐oligosaccharide (GOS) or fructo‐oligosaccharide (FOS), that beneficially and selectively colonize and stimulate the activity of one or more of a limited number of beneficial, health‐promoting, gut microbiota species.^11^ Evidence from multiple randomized controlled trials (RCTs) with up to 5‐year follow‐up suggests prebiotics reduce the incidence and promote the resolution of atopic symptoms,^2^, ^12^, ^13^, ^14^, ^15^, ^16^ reduce the incidence of atopic dermatitis (AD) in infants at risk of allergy,^14^, ^15^, ^16^ improve allergic disease and lessen the progression toward other atopic conditions, a phenomenon known as the “allergic march.”^15^, ^16^, ^17^, ^18^ Probiotics are health‐promoting, non‐pathogenic microorganisms that inhabit the gut such as *Bifidobacterium* (one of the first colonizers of the breast‐fed infant gut and prominent in breastmilk), which beneficially exert immune maturation and stimulatory actions,^19^, ^20^, ^21^, ^22^, ^23^, ^24^ stimulation of gut integrity and modulation of gut microbiota.^19^, ^25^, ^26^, ^27^ Evidence from RCTs has demonstrated improvement of allergic symptoms of infants with atopic dermatitis (AD) supplemented with probiotics^3^, ^19^, ^25^, ^28^, ^29^, ^30^, ^31^ and the use of *Bifidobacterium* and *Lactobacillus* probiotic strains has specifically been shown to significantly improve allergic symptoms^19^, ^29^, ^30^, ^31^, ^32^, ^33^ and intestinal inflammatory markers in those with allergy.^28^, ^34^ The wide‐ranging benefits of pre‐ and probiotics on atopic symptoms and immune function that promote a healthy gut microbiota, more akin to a breastfed infant, appear to be augmented when specific prebiotic compounds selectively and synergistically stimulate the colonization of specific probiotic strains in the host; this pairing is otherwise termed “synbiotics.”^35^, ^36^, ^37^

As mounting evidence suggests that healthy gut microbiota can help improve the “allergic march,” focus around “active” management of food allergy has become of greater interest, as opposed to avoiding food allergens alone.^3^, ^8^, ^26^, ^38^, ^39^ An “active” management approach is one that adopts early introduction of other food allergens, active monitoring, desensitization protocols, and pre‐ and probiotic use.^38^ The window of opportunity to manipulate the diet during infancy provides an easy, noninvasive method of modulating long‐term immuno‐related health outcomes.

The presence of dysbiosis has been confirmed in infants with cow's milk allergy (CMA) which is the most common type of food allergy.^40^, ^41^ Infants with CMA present with a range of symptoms involving the gastrointestinal tract (such as vomiting, reflux), the respiratory system (such as wheeze), and the dermatological system, as CMA often coexists with AD and eczema.^42^ CMA can be differentiated by either an IgE mediated response (immediate onset symptoms) and non‐IgE mediated response (delayed onset symptoms).^41^ Treatment for all infants with CMA involves the dietary exclusion of cow's milk and if breastfed, involves exclusion of cow's milk from the maternal diet, or use of a suitable hypoallergenic formula in those unable to be breastfed.^43^ Both the diagnosis and management of CMA present a significant burden to the healthcare system, in terms of the use of healthcare resources and costs (associated with hospital admissions, appointments with healthcare professionals, and prescriptions for medications).^44^, ^45^

A considerable evidence base and current guidelines for formula‐fed infants with CMA recommend the use of extensively hydrolyzed formulae (eHF) which are nutritionally complete as the first‐line choice.^43^, ^46^, ^47^, ^48^, ^49^, ^50^, ^51^ Although the majority of infants with CMA have a resolution of symptoms with eHF, some do not, or they develop worsening symptoms and may require amino acid‐based formulae (AAF). Specific components of hypoallergenic formulae, such as the addition of pre‐ or probiotics vary and only a few clinical guidelines for the management of CMA recommend their use as the evidence is still emerging.^50^, ^51^, ^52^ There are some prebiotic‐ or probiotic‐containing hypoallergenic formula commercially available for infants with CMA in the UK, Europe, and the United States, but few with a synbiotic combination. Recent systematic reviews on specialized hypoallergenic formula use in infants with CMA have concluded that the use of probiotic only‐containing eHF (*Lactobacillus GG*) may lead to the earlier acquisition of tolerance to cow's milk, whilst the use of synbiotic‐containing‐AAF (prebiotics and *Bifidobacterium breve* M‐16V) may lead to fewer infections, medications, hospital admissions, and subsequent cost savings, as the composition of gut microbiota is improved, analogous to that of breastfed infants.^44^, ^45^ Additionally, high‐quality evidence from RCTs on the provision of either prebiotic components (such as GOS and FOS) or probiotic components (such as *Bifidobacterium breve, Lactobacillus Rhamnosus*, and *L. reteri* and *Lactobacillus GG*) in specialized infant formula has shown significant improvements in gut levels of *Bifidobacterium*, rates of infections, gastrointestinal symptoms, allergic symptoms and AD in allergic infants or those at high risk of atopy, after long term follow‐up.^12^, ^14^, ^15^, ^16^, ^25^, ^26^, ^53^

As multiple probiotic strains exist, evidence comparing various strains in the allergic response identified that *Bifidobacterium breve* M‐16V, originally isolated from breastmilk, was the most effective strain at reducing inflammation.^54^, ^55^, ^56^ *Bifidobacterium breve* M‐16V forms part of a specific synbiotic blend with short‐chain GOS, long‐chain FOS in a specialized infant AAF, which has been shown to significantly improve beneficial gut microbiota species and gut health biomarkers, significantly reduce the number of infections and medication use for infections, and gastrointestinal issues, compared to controls (AAF without synbiotics), in several RCTs in infants with CMA.^57^, ^58^, ^59^ Furthermore, several other clinical studies have found that the *Bifidobacterium breve* M‐16V strain demonstrates efficacy as well as safety in healthy infants and those with atopy.^30^, ^60^, ^61^, ^62^, ^63^

A similar synbiotic‐containing eHF (SeHF), with a specific combination of short‐chain GOS, long‐chain FOS and *Bifidobacterium breve* M‐16V has been assessed in various clinical studies in infants with AD demonstrating similar results, with improvements in the balance of beneficial and pathogenic gut microbiota species, atopic and gastrointestinal symptoms.^35^, ^36^, ^64^, ^65^, ^66^ However, little real‐world clinical evidence exists for the use of synbiotic‐containing eHF in infants with CMA.

The aforementioned SeHF is a whey‐based, specialized infant, hypoallergenic formula intended for the dietary management of CMA in infants. The extensively hydrolyzed formulation contains short‐chain GOS and long‐chain FOS (prebiotics), and *Bifidobacterium breve* M‐16V (probiotics). These pre‐ and probiotic strains have been expressly selected due to their ability to work together in combination as synbiotics, the lack of cow's milk used at source or in processing/production, and the nonallergenic properties of the ingredients, specifically *Bifidobacterium breve M‐16V*. As previously mentioned, this synbiotic combination has been extensively tested for hypoallergenicity, safety, and growth.^12^, ^14^, ^15^, ^16^, ^35^, ^36^, ^58^, ^66^, ^67^ As little real‐world clinical evidence exists for the use of this synbiotic‐containing eHF in infants with CMA, and as clinically meaningful outcomes, including gastro‐intestinal tolerance, AD symptoms, growth, care‐giver burden, and healthcare use have not been extensively studied, research is needed to fill these gaps and inform clinical practice. Therefore, the aim of this small, single‐arm, pilot study was to evaluate the impact of this synbiotic‐containing SeHF in infants with CMA over a 4‐week intervention period, on the clinical symptoms of CMA including gastrointestinal (GI) tolerance and AD, dietary intake, and growth, whilst exploring the acceptability and impact of SeHF on caregiver quality of life. Hospital‐related healthcare use (including hospital visits and prescriptions of medications) was measured in a follow‐up phase to explore the health‐economic impact of SeHF usage in the longer term (up to 6 months after SeHF initiation).

## 2 MATERIALS AND METHODS

### 2.1 Study design and setting

The study was a prospective, single‐arm, longitudinal, interventional, multicentre, 31‐day study. After the baseline visit, infants undertook a 3‐day baseline period whilst continuing their current formula, before the 28‐day intervention period. However, for infants with a clinical requirement to immediately start the study product (SeHF) after the baseline visit (i.e., not currently receiving suitable formula or symptomatic), the 3‐day baseline period was omitted, and infants commenced the SeHF for the 28‐day intervention period. Those who completed the intervention were invited to participate in the follow‐up phase of the study, in which hospital visits and hospital prescriptions of medications were retrospectively collected from hospital medical records and caregivers, during the 6‐months before, and 6‐months after SeHF initiation.

The study was undertaken between October 2017 and November 2020 in nine specialist healthcare centers for the management of CMA, across the UK. Infants were recruited from pediatric nutrition and dietetic outpatient hospital clinics or community services. Ethical approval was obtained from the UK National Health Service (NHS) Research Ethics Committee (London ‐ City & East Research Ethics Committee; 17/LO/1711) and local NHS Research & Development departments reviewed and approved the study for local conduct. The study was registered on Clinical Trials.gov (Identifier: NCT03874104), conducted in line with Good Clinical Practice and the Declaration of Helsinki, and all caregivers of patients recruited were required to provide written informed consent.

### 2.2 Eligibility criteria

Infants were deemed eligible to take part in the study if they were aged <13 months; currently using or requiring an eHF for the dietary management of CMA and expected to receive at least 25% of their energy intake from the SeHF. Infants were excluded from the study if they had severe CMA which required management with an AAF; were exclusively breastfed; were born <37 weeks gestation and of less than 1 month corrected age at time of screening; had primary lactose intolerance; had a history of poor tolerance to whey‐based eHFs; had major hepatic or renal dysfunction; required parenteral nutrition; were receiving postpyloric enteral tube feeding; participated in other clinical intervention studies within 1 month of recruitment, or; if the investigator had concern around the willingness/ability of the caregiver to comply with the protocol and/or study requirements. Written informed consent was obtained from the caregiver of the infant. Eligibility for the follow‐up study phase included infants who had completed the 28‐day intervention with SeHF. Infants who had been subsequently identified as requiring management with AAF, and infants for whom healthcare use data 6‐months before, and after SeHF initiation was unable to be extracted from the hospital medical records, were excluded.

### 2.3 Intervention

All infants received the SeHF (Aptamil Pepti Syneo, Nutricia Ltd.) for the 28‐day intervention period. The SeHF was a hypoallergenic and nutritionally complete eHF, intended for the dietary management of CMA in infants from birth either as a sole source of nutrition or as a supplement alongside breastfeeding and/or complementary foods as appropriate. The SeHF provided 66 kcal, 1.6 g protein, 0.8 g short‐chain GOS, and long‐chain FOS per 100 ml (when reconstituted at the recommended concentration (13.8% w/v)) and 1.2 × 10^8^ CFU/g of *Bifidobacterium breve* M‐16V. Investigators provided each infant an individualized daily minimum target volume of the SeHF, with the recognition that intake would be variable, particularly for infants mixed‐fed (breastmilk and formula) and/or approaching complementary feeding.

## 3 OUTCOMES

### 3.1 Primary outcome

#### 3.1.1 Gastrointestinal tolerance

Gastrointestinal tolerance was recorded at baseline and end of intervention using a standardized questionnaire, to indicate severity (none, moderate, mild, or severe) of vomiting, nausea, abdominal pain/discomfort, bloating, burping, flatulence, diarrhea, and constipation. In addition, daily stool frequency (number per day) and stool consistency (Bristol Stool Form Scale for Children^68^; type 1 (separate hard lumps, hard to pass); type 2 (sausage‐shaped, lumpy); type 3 (sausage shaped with cracks); type 4 (sausage‐shaped, smooth and soft); type 5 (soft blobs with clear cut edges, passed easily); type 6 (fluffy pieces with ragged edges, a mushy stool); type 7 (watery stool, no solid pieces, entirely liquid) were also recorded at the same time points. Investigators were also asked to record if they were satisfied with SeHF tolerance at the end of study.

### 3.2 Secondary outcomes

#### 3.2.1 Atopic dermatitis and other atopic symptoms

The presence and severity of AD symptoms were recorded using a validated assessment tool (Patient Reported SCORing of Atopic Dermatitis [PO‐SCORAD^©^])^69^ at baseline, intervention Day 7 and 28. This rated severity (from none to extreme) of dryness, erythema, edema, oozing, scratches, skin thickening, as well as indicating sleep quality and crying frequency on visual analog scales (100 mm; ranging from 0 [none] to 10 [unbearable]). The numeric value from the tool indicated a score ranging from 0 (no skin affected and no symptoms) to 103 (entire body affected and symptoms extreme). Furthermore, atopic symptoms (wheeze, rhinitis, and itchy/watery eyes), crying, and sleep quality were assessed at baseline and intervention Day 7, 14, 21, and 28 using visual analog scales (100 mm) ranging from 0 (no symptoms) to 10 (worst imaginable symptoms).

#### 3.2.2 Caregiver quality of life

Caregiver measures of the physical and emotional quality of life (related to the burden of their child's allergy day to day, in social settings and in future) were assessed via a standardized, validated questionnaire, Food Allergy Quality of Life‐Parental Burden (FAQL‐PB),^70^, ^71^ at baseline and end of the intervention. The FAQL‐PB is a 17‐item questionnaire with a 7‐point Likert scale (from not troubled (0), hardly troubled at all (1), somewhat troubled (2) moderately troubled (3), quite a bit troubled (4), very troubled (5), or extremely troubled (6)), where scores range from 0 (no burden) to 102 (extreme burden). Overall score was used to reflect impact of infant allergy on the total quality of life for caregivers.

#### 3.2.3 Acceptability

Caregivers were asked to complete an acceptability questionnaire at baseline and at the end of intervention. This questionnaire used 5‐point Likert scales (from strongly agree (1), agree (2) don't know (3), disagree (4) or strongly disagree (5)) to assess ease of preparation and use, infant enjoyment, and overall liking of their baseline formula and SeHF.

#### 3.2.4 Dietary intake and dietetic goals

Dietary intake was recorded at baseline and end of intervention, using comprehensive 24‐h dietary recall conducted by the investigators. Dietary intake data were analyzed using a dietary analysis program (Nutritics v5.026 (Research Edition), Dublin) to estimate energy and macronutrient intakes. Due to the difficulty in assessing the nutritional composition of breastmilk, this was excluded from the nutritional analysis. Intake of infants' baseline formula and the SeHF were reported daily (in ml) throughout the study by the caregivers. At the end of the study, this was compared to the daily minimum target volume prescribed by the investigators to assess compliance, and overall achievement of expected intake of the SeHF was also recorded. Dietetic goal(s) related to the introduction of the SeHF over the intervention period, were set for each infant by investigators at baseline. These could relate to symptom management, intake, growth, GI tolerance, or any other relevant outcome. Achievement of these goals was recorded at the end of study.

#### 3.2.5 Growth

At baseline and end of the intervention, standardized methods for supine length (recorded to nearest 0.1 cm), weight (calibrated infant weighing scale with tray (accurate to 0.01–0.02 kg) recorded to nearest 0.1 kg), and head circumference (measured with a slotted nonstretchable tape, recorded to nearest 0.1 cm) were used to measure infant growth by investigators. These measures were used to determine growth centiles (plotted on UK‐WHO growth charts 0–4 years) and *z*‐scores were calculated using LMS growth macros for excel (based on UK‐WHO 2006 growth reference data for children 0–4 years old).^72^

#### 3.2.6 Safety

During the study, serious adverse events were monitored and recorded by investigators, indicating severity and relatedness to the use of the SeHF.

#### 3.2.7 Hospital‐related healthcare use

Of the infants included in the follow‐up phase, the following data were retrospectively extracted from hospital medical records, 6 months before and 6 months after SeHF initiation:

1) Hospital visits (elective and emergency admissions and attendances including outpatients).
2) Hospital medication prescriptions (medication prescribed in hospital settings including antibiotics, inhalers, anaphylaxis‐adrenaline, and those for the management GI and dermatological conditions).

To capture hospital visits and medication prescriptions outside of the study site where the infant was recruited (as such events would not be recorded in the hospital medical records at the study site), caregivers were asked to recall their child's healthcare use outside of the study site. This data was added to the data extracted from the medical records. For each 6‐month time period, hospital visits and medication prescriptions were recorded as the mean total number across the sample and the % of infants with ≥1 hospital visit or medication prescription.

### 3.3 Statistical methods

Data were analyzed per the protocol and statistical procedures were performed using a statistical analysis package (SPSS v24, IBM Corp.). Descriptive data (means, percentages, standard deviations, and ranges) are provided where applicable. Significance was assumed at an α level of *p* < .05. For continuous data (growth, compliance, and nutrient intake), normal distribution was verified with the use of the Kolmogorov–Smirnov normality test before statistical analysis. Paired samples *t* tests were used for comparisons of two‐time points (baseline vs. end of study) for growth, compliance, nutrient intake, and caregiver quality of life (FAQL‐PB scores). FAQL‐PB scores were also analyzed per question (as well as overall) to understand if there were any specific differences. Nonparametric tests (Wilcoxon signed‐rank and Friedman tests) were used to analyse ordinal data (GI tolerance, AD severity (PO‐SCORAD^©^), atopic symptoms, acceptability, and hospital‐related healthcare usage). AD severity was also analyzed based on baseline PO‐SCORAD^©^ score quartiles (upper quartile range 17.5–86.3 [*n* = 6]; third quartile range 8.8–14.1 [*n* = 6]; second quartile range 5.6–8.5 [*n* = 6]; lower quartile range 0.1–5.4 [*n* = 6]). Patients with baseline score of 0 (i.e. no signs of AD) were excluded from this analysis. For all outcomes, analysis was also performed by comparison to historic formula use at baseline (specifically those on probiotic‐containing eHF [*n* = 13] or prebiotic containing eHF [*n* = 12]).

The sample size was calculated based on results from a similar study (ClinicalTrials.gov Identifier: NCT02915510) showing mean 86.2% (SD 11.6) absence of gastro‐intestinal symptoms; assuming the power of 80% and an *α* level of .05, for noninferiority, the minimum detectable effect of 5 (noninferiority margin 0), which provided a sample size of 34. Therefore, a sample size of 40 patients was deemed suitable for this pilot study, allowing for dropouts.

## 4 RESULTS

### 4.1 Baseline descriptive data

**Figure 1:**
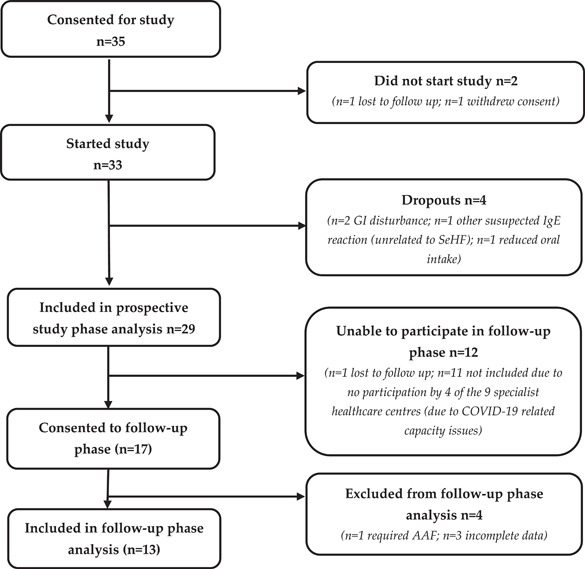
Flow chart of patient recruitment and study participation.

Of the 35 infants that met the study criteria and provided consent, a total of 33 infants began the study (*n* = 1 lost to follow‐up; *n* = 1 withdrew consent, Figure 1). Five infants omitted the 3‐day baseline period after the baseline visit, due to a clinical requirement to start the SeHF immediately. Baseline and end of study data were collected in 29 patients (*n* = 28 completed full study; *n* = 1 completed 23 days (20 days on SeHF)) and were included in this analysis. Four patients dropped out of the study within the first 2 weeks due to: GI disturbance (*n* = 2; 4–11 days on SeHF); other suspected IgE reaction (*n* = 1, related to use of new baby wipes; 1 day on SeHF); reduced oral intake of SeHF (*n* = 1; 2 days on SeHF) (see Figure 1). Of the 29 infants included in the final analysis of the prospective study (see Table 1), mean age at baseline was 30.8 weeks ([SD 11.0] [range 13.4–53.4]), all infants were categorized as non‐IgE CMA by the investigators (mean age at diagnosis 13.0 weeks ([SD 12.2] range 2.4–52.1]). Common secondary diagnoses included gastro‐esophageal reflux disease (GORD), eczema, and constipation. Severity of AD (measured by PO‐SCORAD^©^) at baseline was relatively low (mean 11.3 [SD 17.2]), as the majority of infants were already receiving an eHF, however, severity varied substantially (range 0–86.3). Most infants had no growth concerns at baseline, however, three were identified with faltering growth. Baseline formula was eHF (*n* = 27) of which, *n* = 13 were probiotic‐containing, *n* = 12 were prebiotic‐containing and *n* = 2 were standard eHF (i.e. no pre‐ or probiotics). Mean time on eHF at baseline was 15.6 weeks ([SD 11.8] [range 4.7–49.3]). Overall, only two infants were mixed‐fed receiving at least some breastmilk as well as formula at baseline. All infants were orally fed, of which 69% had started complementary feeding at baseline (*n* = 20). Seventeen infants were invited to participate in the follow‐up phase (Figure 1), but only 13 were eligible for the follow‐up phase (mean age at the time of inclusion 29.2 months [SD 6.1] [range 11.5–35.9]).

**Table 1:** Baseline demographics of infants included in the study (*n* = 29).

Baseline characteristic		Result
Age (weeks)	Mean (SD) [range]	30.8 (11.0) [13.4–53.4]
Age of CMA diagnosis (weeks)	Mean (SD) [range]	13.0 (12.2) [2.4–52.1]
Sex	Male: Female	16: 13
Length (cm)	Mean (SD)	68.2 (5.4)
Weight (kg)	Mean (SD)	7.9 (1.4)
Head circumference (cm)	Mean (SD)	43.6 (2.2)^a^
PO‐SCORAD^©^ (baseline visit)	Mean (SD) [range]	11.3 (17.2) [0–86.3]
Baseline formula	*N*	Probiotic‐containing eHF *n* = 13
		Prebiotic‐containing eHF *n* = 12
		Standard eHF (no prebiotic or probiotic) *n* = 2
		Standard infant formula *n* = 2
Mean time on baseline eHF (weeks)	Mean (SD) [range]	15.6 (11.8) [4.7–49.3]
Type of CMA	*N*	non‐IgE mediated *n* = 29
Comorbidities	*N*	GORD *n* = 10
		Eczema *n* = 6
		Constipation *n* = 2
		Tree nut allergy *n* = 1
		Peanut allergy *n* = 1
		Sesame sensitization *n* = 1
Medications (*indication*)	*N*	Anti‐GORD *n* = 16 (GORD *n* = 16)
		Anti‐biotic *n* = 4 (tonsillitis *n* = 2 & conjunctivitis *n* = 2)
		Emollient *n* = 2 (eczema *n* = 2)
		Laxative *n* = 3 (constipation *n* = 3)
		Anti‐fungal *n* = 1 (nappy rash *n* = 1)
		Corticosteroid *n* = 3 (eczema *n* = 2; AD *n* = 1)
		Bronchodilator *n* = 1 (asthma *n* = 1)
		Anti‐colic *n* = 1 (colic *n* = 1)

Abbreviations: AD, atopic dermatitis; CMA, cow's milk allergy; eHF, extensively hydrolyzed formula; IgE, immunoglobulin‐E; GORD, gastro‐esophageal reflux disease; PO‐SCORAD^©^, patient‐reported scoring of atopic dermatitis; SD, standard deviation.

a *n* = 1 missing data

### 4.2 Main results

#### 4.2.1 Gastrointestinal tolerance

**Figure 2:**
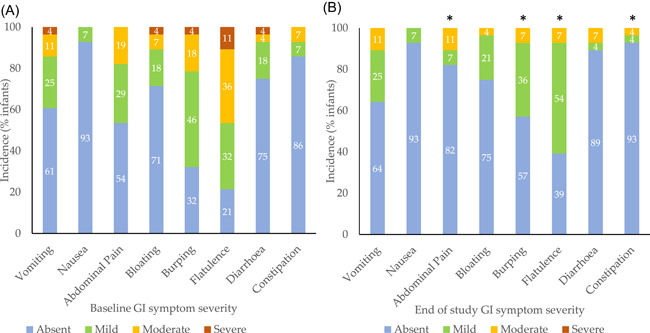
Incidence (% of infants) and severity of gastrointestinal symptoms at baseline (A) and end of study following use of SeHF (B). Severity ratings are shown as absent (blue), mild (green), moderate (yellow), or severe (red). A significant difference in ratings between baseline and end of study (*p < 0.05). SeHF synbiotic‐containing, whey‐based extensively hydrolyzed formula

By the end of the study, incidence and severity of abdominal pain, flatulence, burping, and constipation significantly improved in infants whilst taking SeHF, and no severe symptoms were recorded at all (compared with 27% severe ratings across all symptoms at baseline) (see Figure 2). Abdominal pain improved in 57% (*n *= 16) of infants (*Z* = −2.972, *p* = .003), with absence of abdominal pain increasing by 39% at the end of study. Burping improved significantly in 46% (*n* = 13) of infants by the end of study (*Z* = −2.321, *p* = .02) with no severe ratings recorded. Flatulence improved significantly in 79% of infants (*Z* = −2.802, *p* = .005), where severe symptoms resolved, and the majority of symptoms were rated as mild (reduced from moderate in 31%) by end of study. Constipation improved significantly in 14% of infants (*Z* = −1.890, *p* = .04), which although largely absent in the majority of patients, further improved by 7% at the end of study (see Figure 2). Other gastrointestinal symptoms including vomiting, nausea, bloating, and diarrhea were largely absent or mild at baseline and remained unchanged in infants whilst taking SeHF (*p* > .05, NS).

Stool frequency remained relatively stable with the use of SeHF (mean stool frequency baseline: 2.4 (SD 1.2) and end of study: 1.9 (SD 1.1), *p* = .12, NS) as did consistency of stools (mean Bristol Stool Form Scale: baseline 5.2 (SD 1.5) and end of study: 4.7 (SD 1.6), *p* = .097, NS). Furthermore, investigators either strongly agreed or agreed that the SeHF was well tolerated in 85% of infants by the end of study. Analysis by baseline eHF type found no differences in gastrointestinal tolerance of SeHF based on the historic use of pre‐ or probiotic‐containing eHF at baseline.

#### 4.2.2 Atopic dermatitis and other atopic symptoms

**Figure 3:**
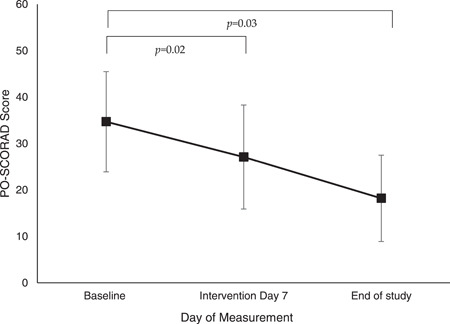
Changes in atopic dermatitis severity (PO‐SCORAD^©^ score) from baseline to intervention Day 7 and end of study following SeHF use in those with severe atopic dermatitis at baseline (*n* = 6). PO‐SCORAD^©^, patient‐reported scoring of atopic dermatitis; SeHF, synbiotic‐containing, whey‐based extensively hydrolyzed formula

Severity of AD was relatively low at baseline (mean PO‐SCORAD^©^: 11.2 (SD 16.9) [range 0–86.3) as the majority were already established on an eHF, although a wide range of severity was observed. Overall PO‐SCORAD^©^ remained stable throughout the study in infants whilst taking SeHF (mean end of study PO‐SCORAD^©^: 7.8 (SD 13.0); *p* = .09, NS). Analysis of infants with the most severe AD at baseline (i.e. those in upper quartile of PO‐SCORAD^©^ [mean PO‐SCORAD^©^: 34.7 (SD 26.5) [range 17.5–86.3]; *n* = 6), demonstrated a significant reduction in AD severity by the end of the study (mean PO‐SCORAD^©^: 18.2 (SD 22.7); *p* = .03) and improvements were seen as early as intervention Day 7 (mean PO‐SCORAD^©^: 27.1 (SD 27.4); *p* = .02) (see Figure 3). There was also a significant improvement detected in the third quartile (see Table 2), but no other significant changes were seen in the lower two quartiles. Atopic symptom severity improved significantly in infants whilst taking the SeHF during the study with reduction in the severity of rhinitis by 41% (baseline mean score: 34.7 (SD 31.2); end of study mean score: 20.4 (SD 27.3); *p* = .048) however improvements were detected as early as intervention Day 7 (mean: 18.2 (SD 25.1); *p* = .019 vs. baseline). Severity of itchy eyes also reduced significantly by 73% at the end of study (baseline mean score: 19.0 (SD 30.4); end of study mean score: 5.2 (SD 11.4); *p* = .021). Severity of crying (baseline mean score: 24.5 (SD 17)), poor sleep (baseline mean score: 33.7 (SD 24.4), and wheeze (baseline mean score: 9.8 (SD 15.1)) were relatively low and remained stable at end of study (*p* > .05, NS). Analysis of atopic symptom severity did not detect any differences based on historic formula use.

**Table 2:** Atopic dermatitis severity measured by PO‐SCORAD^©^ from baseline to intervention Day 7 and end of study following use of SeHF (Mean (SD)), based on baseline PO‐SCORAD^©^ score quartiles.

Baseline PO‐SCORAD^©^ quartile [range]	Baseline PO‐SCORAD^©^	Intervention Day 7 PO‐SCORAD^©^	*p* *	End of study PO‐SCORAD^©^	*p* *
Upper [17.5–86.3] (*n* = 6)	34.7 (SD 26.5)	27.1 (SD 27.4)	.02	18.2 (SD 22.7)	.003
Third [8.8–14.1] (*n* = 6)	12.6 (SD 3.7)	11.8 (SD 10.5)	.838	7.6 (SD 5.9)	.033
Second [5.6–8.5] (*n* = 6)	5.9 (SD 1.9)	4.9 (SD 8.3)	.777	8.6 (SD 13.3)	.622
Lower [0.1–5.4] (*n* = 6)	2.1 (SD 1.9)	5.0 (SD 6.1)	.293	3.0 (SD 3.3)	.312

Abreviation: PO‐SCORAD^©^, patient‐reported scoring of atopic dermatitis; SD, standard deviation.

* Compared with baseline.

#### 4.2.3 Caregiver quality of life

Caregivers reported significant improvements in quality of life by the end of study after use of SeHF. Scores from the FAQL‐PB questionnaire demonstrated significant reduction by 26.7% from baseline (mean: 30.9 (SD 23.1)) to end of study mean: 22.6 (SD 19.3), *p* = .004). There were no differences following analysis according to baseline eHF type, nor by scores to each specific question in the FAQL‐PB questionnaire.

#### 4.2.4 Acceptability

Perceived enjoyment significantly improved at the end of the study with SeHF when compared to baseline formula (from 82% at baseline to 91% end of study, *p* = .02). Overall, 92% of caregivers strongly agreed or agreed the SeHF was acceptable compared to baseline formula (*p* = .06, NS). No differences in the ease of preparation and use of formula were reported between baseline formula and SeHF by the end of study (*p* = .083, NS). Furthermore, analysis of those on prebiotic‐containing eHF at baseline (requiring different preparation to probiotic‐containing formulae) confirmed that there were no differences in ease of preparation and use of SeHF (*p* = .183, NS).

#### 4.2.5 Dietary intake and dietetic goals

Mean total energy and macronutrient intake from formula and the complementary diet remained stable between baseline and the end of study (Energy: 704 kcal/d [SD 186] vs. 699 kcal/d [SD 194], *p* = .891 and Protein: 21.8 g/d [SD 9.5] versus 21.6 g/d [SD 9.9], *p* = .949, respectively). Percentage energy from fat and carbohydrate from SeHF was similar to baseline formula. There was a difference in percentage energy from protein (*p* = .046), which was likely due to the differences in protein content (mean 1.78 g/100 ml baseline formula vs. 1.6 g/100 ml SeHF) however overall protein intakes were unchanged during the study (*p* = .949, NS). Mean intake of SeHF during the study was 615 ml/d (SD 223) and was maintained at levels similar to the intake of baseline formula (mean 637 ml/d (SD 253), *p* = .375, NS). Mean daily intake of SeHF was 120% (SD 56) of patient's target minimum daily volume, demonstrating good compliance to the SeHF overall. Investigators reported that 26 (96%) patients achieved expected intake (for n = 1 who did not achieve expected intake, the investigator reported this was appropriate due to increased complementary feeding). All investigators set one or more dietetic goals for each infant on the study which were related to symptoms of CMA, growth, intake of SeHF, obviating need for AAF, and provision of synbitoics to the gut microbiome. By the end of the study 90% of infants (*n* = 26) met all their dietetic goals and 7% (*n* = 2) met at least one. Furthermore, 80% of infants remained on the SeHF following study completion.

#### 4.2.6 Growth

By the end of the study, a significant increase in all absolute anthropometric measurements (mean length, weight, and head circumference) was observed (Table 3; *n* = 28). Furthermore, there were significant increases in measures of relative growth (centiles and z scores) for all measurements (length, weight, and head circumference), despite a stable energy and protein intake over the study. Weight for length centiles and z‐scores improved, although non‐significantly, by the end of the study (NS) (see Table 3). Investigators reported that 100% of infants grew as expected over the study period. Of the three infants identified with faltering growth at recruitment, this was corrected for one infant by the end of study.

**Table 3:** Growth measures at baseline and end of study (mean (SD))^a^

Measure		Baseline mean (SD)	End of study mean (SD)	*p*
Length	cm	68.3 (5.5)	70.6 (4.9)	<.001
	centile	50.1 (29.8)	58.8 (26.9)	<.001
	*z*‐score	−0.05 (1.1)	0.29 (0.94)	<.001
Weight	kg	8.0 (1.46)	8.5 (1.4)	<.001
	centile	46.0 (24.6)	54.1 (23.4)	<.001
	*z*‐score	−0.09 (0.72)	0.15 (0.71)	<.001
Head circumference	cm	43.5 (2.2)	44.5 (2.01)	<.001
	centile	53.7 (29.4)	61.3 (25.4)	.031
	*z*‐score	0.10 (0.92)	0.41 (0.92)	.011
Weight for length	centile	51.1 (29.7)	53.2 (28.9)	.469
	z‐score	0.07 (0.93)	0.14 (0.94)	.529

Abbreviation: SD, standard deviation.

^a^ *n* = 28, *n* = 1 missing data.

#### 4.2.7 Safety

No serious adverse events were reported over the duration of the study and no safety concerns were reported with the use of SeHF. Overall, 10 adverse events were recorded in 7 infants (infection/virus [*n* = 5]; GI disturbance [*n* = 2]; facial rash [*n* = 1]; loose stools and teething [*n* = 1]; teething [*n* = 1]). The majority of adverse events (9 out of 10) were reported as mild‐moderate, and only 3 out of 10 were deemed probably related to the SeHF. Of these, two were reported in the same infant which involved loose stools and teething, however, this infant completed the study with no issues and continued with the SeHF after intervention. The remaining adverse event was in one infant who terminated the study due to pain in the gut, loose stools with mucus, crying, and sneezing.

#### 4.2.8 Hospital‐related healthcare use

Significant reductions in the mean number of overall hospital visits required by infants were observed in the 6 months after SeHF initiation compared with the 6 months prior (Table 4). The largest decrease was apparent for elective hospital attendances (mean 2.77 [SD 2.01] pre‐SeHF vs. mean 1.46 [SD 1.45] post‐SeHF, *p* = .019). A reduction in the incidence of any type of hospital visits was also observed, although this did not reach significance (*p* = .083). The incidence and mean number of overall hospital medication prescriptions were significantly lower amongst infants in the 6‐month after SeHF initiation compared with the 6 months prior (Table 4). This was most apparent in prescriptions for gastrointestinal conditions (46.2% vs. 7.7% prevalence pre‐ and post‐SeHF, respectively, *p* = .025).

**Table 4:** Incidence (%) and mean number (SD) of hospital visits and hospital medication prescriptions in the 6 months before (pre‐SeHF), and 6 months after (post‐SeHF), SeHF initiation, amongst infants included in the follow‐up phase (*n* = 13).

Healthcare type		Mean number (SD)			Incidence (%)		
		6 months pre‐SeHF	6 months post‐SeHF	*p*	6 months pre‐SeHF	6 months post‐SeHF	*p*
Hospital visits	Elective hospital attendance or admission	2.77 (2.01)	1.46 (1.45)	.019	100	76.9	.083
	Emergency hospital attendance or admission	0.69 (1.18)	0.38 (0.96)	.279	38.5	15.4	.180
	Overall hospital visits	3.46 (2.60)	1.85 (2.30)	.005	100	76.9	.083
Hospital medication prescriptions	Antibiotic	0.08 (0.28)	0.08 (0.28)	–	7.7	7.7	–
	Inhaler or anaphylaxis adrenaline	0	0	–	0	0	–
	GI‐related	1.38 (2.14)	0.08 (0.28)	.043	46.2	7.7	.025
	Dermatological‐related	0.92 (3.04)	0	.180	15.4	0	.157
	Overall medication prescriptions	2.38 (4.87)	0.15 (0.56)	.027	53.8	7.7	.014

Abbreviations: GI, gastrointestinal; SD, standard deviation; SeHF, synbiotic‐containing extensively hydrolyzed formula.

## 5 DISCUSSION

The primary aim of this small, single‐arm, pilot study was to evaluate the impact of SeHF over 4 weeks in infants with CMA on clinically meaningful outcomes. Findings included significant improvements in GI tolerance over time (primary outcome), reductions in atopic symptoms and AD in those most severely affected at baseline, growth, high caregiver acceptability, and improved parental quality of life.^73^, ^74^, ^75^ Furthermore, hospital visits and hospital prescriptions for medications were significantly reduced after 6 months amongst infants with CMA initiated on SeHF. Although this is a small study, without a control group, these data contribute to the understanding of the role of synbiotics in CMA management.

Primary outcome results demonstrated that infants, historically taking a nonsynbiotic eHF (before study), had significant improvements in the incidence and severity of abdominal pain, burping, flatulence, and constipation over time whilst taking SeHF. The mean duration over which infants had been receiving an eHF at baseline in this study was 15.6 weeks (SD 11.8; range 4.7–49.3), which is longer than the average time required for symptom resolution reported in the literature (range 4.8–13.6 weeks).^44^, ^76^ This suggests that infants had sufficient time on an eHF at baseline (before the study), indicating that the GI benefits observed were due to the use of the SeHF, although this is difficult to interpret without a control group. Furthermore, the average time from CMA diagnosis to study enrollment was around 4.5 months, and existing evidence suggests outgrowth (i.e. observed improvements) of CMA is around 1–2 years.^77^, ^78^ As such, observations in this cohort of infants alludes to the benefits of the synbiotic formula used, specifically the combination of pre‐ and probiotic ingredients GOS, FOS, and *Bifidobacterium breve M‐16V*, which is in line with results in other similar studies.^57^, ^58^, ^59^, ^67^ Moreover, the probiotic added to the SeHF in this study (*Bifidobacterium breve M‐16V*) is supported by a wealth of GI tolerance data in both healthy and atopic infants receiving an AAF with the same probiotic strain.^12^, ^14^, ^15^, ^16^, ^35^, ^36^, ^66^, ^67^ Gastrointestinal symptoms are one of the commonest symptoms reported in CMA and they can have a large impact on the patient and parent/caregiver. Improvements in GI symptoms over time as observed in this study, may represent a clinically important outcome, and improvement in patient condition and parent/carer burden, although this is difficult to interpret without a control group.

Although AD severity (PO‐SCORAD^©^) did not change in the total study group, those with the most severe symptoms (*n* = 12) observed significant improvements over time. The severity of the atopic symptoms rhinitis and itchy eyes also improved significantly over time, but other symptoms did not show any changes. Considering that symptom severity was low at the beginning of the study, most likely due to established non‐synbiotic containing eHF use, the further reduction in AD symptoms is interesting, although difficult to interpret without a control group. Similar results have also been found in other studies on GOS, FOS and *Bifidobacterium breve M‐16V* demonstrating improved AD severity in non‐CMA infants taking the same SeHF used in this study.^12^, ^15^, ^16^, ^30^, ^36^ Further investigation may discover changes in the use of medications for the control of atopic symptoms long term. Improvements in AD symptoms in patients with CMA represent a substantial improvement in patient condition and parent/caregiver burden.

It has been well established in the literature that those caring for individuals with food allergies suffer from a lower quality of life due to the emotional and social impact of living with the allergies, and caregivers typically report high‐stress levels, high levels of anxiety, and a high impact of the child's allergy on normal life.^79^ Even though caregiver quality of life was relatively high at baseline (denoted by lower score) compared to the literature, further significant improvements in caregiver quality of life over time were observed after infants had been taking the SeHF, at the end of study,^70^, ^71^ although this is difficult to interpret without a control group. Despite the relatively short time frame of the intervention period (4 weeks), and that the majority of infants in the study were already established on an eHF at recruitment, the improvement in caregiver quality of life was notable, although difficult to interpret without a control group. It could be hypothesized that this observation was related to the improvements in GI and atopic symptoms found in this study, as these symptoms appear most likely to impact caregiver burden/quality of life.

The importance of adequate nutritional intake is paramount in any infant, as well as those with CMA, particularly in view of the heightened risk of impaired growth^80^ especially if CMA persists into childhood.^81^ The positive infant z‐scores and centiles for weight, length and head circumference observed by the end of the study were apparent despite a stable overall energy and protein intake in comparison to baseline. Whilst the results of this study are difficult to interpret without a control group, evidence from RCTs using the same SeHF has confirmed good growth (when compared with controls or healthy infants).^12^, ^14^, ^15^, ^16^, ^35^, ^36^, ^58^, ^66^, ^67^

The SeHF was found be to highly acceptable by caregivers of infants although it required different preparation than nonprobiotic‐containing eHF (due to the presence of probiotic bacteria). In this study, 100% of caregivers found the SeHF as easy to use as baseline formula (which included *n* = 16 on non‐probiotic eHF). Furthermore, subgroup analysis of those on non‐probiotic (prebiotic‐containing) eHF at baseline confirmed that there were no differences in ease of use with SeHF (*p* = .183, *NS*) which supports the practicality of using the synbiotic‐containing formula. Clinical guidelines recognize palatability as an important consideration when choosing suitable formula for the dietary management of CMA,^82^ as many infants will have developed taste preferences by the time of diagnosis (particularly for older infants).^83^ Evidence demonstrates good palatability has the potential to reduce formula wastage and healthcare costs, and that whey‐based, lactose‐containing eHFs are more palatable than casein‐based and/or lactose‐free eHFs, as well as being safe for use.^84^, ^85^ The SeHF in this study is based on the same whey‐based protein hydrolysate with lactose, as other existing eHF.^83^ Acceptability outcomes in this study demonstrated that caregivers reported greater perceived enjoyment of the SeHF when compared to the baseline formula (which predominantly did not contain lactose [55%]).

The management of CMA is associated with substantial healthcare costs^44^, ^45^ and the significant reduction in hospital visits and prescriptions for medications observed in this study have potentially important health‐economic implications although are difficult to interpret without a control group. Similar findings have been reported in a systematic review of synbiotic‐containing AAFs amongst infants with CMA which reported significant reductions in infections and potential cost savings of > £330 per infant, aswell as symptom resolution and healthy growth.^86^ These clinical and health‐economic benefits were hypothesized to be the result of the concurrent changes in the gut microbiome composition becoming closer to that of a healthy breast‐fed infant. The findings from the present study add to the evidence base, showing that the addition of synbiotics to eHF could result in clinical benefits that lessen medication requirements and hospital visits amongst infants with CMA. However, further controlled trials that include prospective data collection of healthcare use from across all settings, in larger samples of infants with CMA, are warranted to confirm these potential health‐economic implications.

Whilst this study makes a valuable contribution to the evidence base for the use of synbiotic‐containing eHF in the management of infants with CMA, the results need to be assessed with extreme caution due to the small sample size and lack of control group. It is therefore not without its limitations. First, this was a small, single‐arm, pilot study, to evaluate the use of a SeHF in infants with CMA. The sample size was based on a power calculation to investigate changes in gastrointestinal tolerance, and improvements were observed. The study was designed to mimic clinical practice with clinically meaningful outcomes, based on the common symptoms and issues relating to CMA management including parental burden. Therefore, all infants identified with CMA by the investigators and being under the care of allergy services were eligible to participate, resulting in a heterogeneous group of infants being included in the study, some with comorbidities and multiple allergies. All infants were identified as having non‐IgE mediated CMA by the investigators, however, the method of identification was not recorded. Therefore, the infants included in the study may have been a mix of IgE and non‐IgE mediated CMA, however, this should not affect the interpretation of the results, and mimics real‐life clinical practice. Furthermore, though infants served as their own control in the baseline period, comparison to a separate control group, as part of a randomized controlled trial, would have allowed for far greater interpretation of outcomes. Second, there was no standardization of CMA management (i.e., type of eHF at baseline, use of milk ladder), and whilst all were following a cow's milk protein‐free diet, four infants were also following a soya free diet (though allergy was unconfirmed). Existing evidence suggests the presence of soya may risk cross‐reactivity in those with CMA and whilst most guidelines do not suggest soya formula first line, even fewer discuss routine exclusion of soya in complementary foods.^50^, ^51^, ^87^ Due to the small number excluding soya in this study, it is unlikely exclusion had an impact on the outcomes seen, however future trial designs should consider differences in CMA management. Third, due to constraints of study setup, fecal analysis was not carried out, therefore changes in the composition of gut microbiota were not investigated, though this has been undertaken in other studies.^57^, ^58^, ^59^ Although this would have been a desirable outcome to collect, outcomes such as GI tolerance and atopic symptoms were prioritized for investigation in this study as more clinically relevant to the management of CMA. Fourth, the study was limited by the short intervention period and small sample size, and spanned over the COVID‐19 pandemic. This restricted access to community‐based infants for recruitment (though one infant was successfully, remotely recruited), and investigator participation was restricted in the follow‐up phase due to clinical priorities related to COVID‐19. Finally, it was only possible to extract healthcare use from hospital medical records during the follow‐up phase, and therefore community healthcare usage such as GP appointments and GP‐initiated prescriptions were not captured which is where a large majority of healthcare usage exists for infants with CMA.^44^ Therefore, it is likely that the healthcare use data collected in this study is underestimated and warrants further investigation with age‐ and sex‐matched controls for comparison in healthy infants and those on other types of hypoallergenic formula for the clinical management of CMA.

## 6 CONCLUSIONS

Overall, the results of this small, single‐arm, pilot study demonstrate that SeHF containing GOS, FOS, and *Bifidobacterium breve M‐16V* enhances the dietary management of CMA infants already well established on eHF, although the results of this study are difficult to interpret without a control group. By the end of the study, significant improvements were observed in abdominal pain, burping, flatulence, constipation, rhinitis, itchy eyes, AD severity in those with most severe symptoms, and growth, whilst caregiver quality of life was also improved. Furthermore, SeHF was associated with reduced hospital‐related healthcare use in the 6 months after initiation in this cohort. This study adds to the evidence highlighting and supporting the clinical and health‐economic benefits of using synbiotic‐containing hypoallergenic infant formula for the dietary management of CMA, although the results of this study are difficult to interpret without a control group. The results in this study are specific to the synbiotic short‐chain GOS, long‐chain FOS, and *Bifidobacterium breve* M‐16V and cannot be extrapolated to other types of synbiotic formulae. Furthermore, controlled, robust research to explore the longer‐term benefits of synbiotics, specifically the blend used in this study, for the clinical management of infants with CMA is required.

## AUTHOR CONTRIBUTIONS

Conceptualization and methodology prospective study phase: Robert M. Browne, Gary P. Hubbard, and Rebecca J. Stratton. Conceptualization and methodology follow‐up study phase: Fiona J. Kinnear and Abbie L. Cawood. Chief investigator: Rebecca J. Stratton. Investigation and acquisition of data: Robert M. Browne, Kiranjit Atwal, Fiona J. Kinnear, Lynne Graham, Sankara Narayanan, Lisa Cooke, Catherine Casewell, Sally‐Ann Denton, Joan Gavin, Ailsa J McHardy, Debbie Evans, Rachel Vallis, Devasmitha Venkataraman, Sarah Donohoe, Victoria Steele, Sonia Armstrong, and Gary P. Hubbard. Formal analysis, Kiranjit Atwal, Robert M. Browne, Fiona J. Kinnear, and G.P.H; Writing—original draft preparation, Kiranjit Atwal. Writing—review and editing, Kiranjit Atwal, Gary P. Hubbard, Fiona J. Kinnear, and Rebecca J. Stratton. Visualization, Robert M. Browne, Kiranjit Atwal, Gary P. Hubbard, and Rebecca J. Stratton. Supervision: Kiranjit AtwalA, Gary P. Hubbard, and Rebecca J. Stratton. Project administration, Robert M. Browne, Kiranjit Atwal, and Gary P. Hubbard. Funding acquisition, Gary P. Hubbard and Rebecca J. Stratton.

## CONFLICTS OF INTEREST

K.A, G.P.H, R.M.B, A.L.C, F.K., and R.J.S. are employees of Nutricia Ltd; L.C, has received payment from Nutricia for previous writing activities; L.C, S. Denton, have received educational grants from Nutricia Ltd. for conference attendance; L.C, S. Denton, have received honorarium from Nutricia Ltd. for conference presentation; have provided scientific advice and expertise for Nutricia Advisory Boards; L.G, C.C, S.N, J.G, A.J.M, D.E, R.V, D.V, S. Donohoe, V.S, and S.A. declare no competing interests with the study funder.

## ETHICAL STATEMENT

The study was approved by the UK National Health Service (NHS) Research Ethics Committee (London‐City & East Research Ethics Committee; 17/LO/1711) and local NHS Research & Development departments reviewed and approved the study for local conduct. The study was registered on Clinical Trials.gov (Identifier: NCT03874104) and conducted in line with Good Clinical Practice and the Declaration of Helsinki. Informed consent was obtained from all parents/carers involved in the study.

## Data Availability

The data that support the findings of this study are available from Nutricia Ltd, UK but restrictions apply to the availability of these data, which were used under license for the current study, and so are not publicly available. Data are however available from the corresponding author upon reasonable request and with permission of Nutricia Ltd and respecting the EU GDPR regulation.
